# Associations between hypertension with reproductive and menopausal factors: An integrated women’s health programme (IWHP) study

**DOI:** 10.1371/journal.pone.0299840

**Published:** 2024-03-25

**Authors:** Laureen Yi-Ting Wang, Win P. P. Thu, Yiong Huak Chan, Susan Logan, Michael S. Kramer, Jane A. Cauley, Eu-Leong Yong

**Affiliations:** 1 Department of Obstetrics and Gynaecology, National University Hospital, National University of Singapore, Singapore, Singapore; 2 National University Heart Centre, National University Hospital Singapore, Singapore, Singapore; 3 Yong Loo Lin School of Medicine, Biostatistics Unit, National University of Singapore, Singapore, Singapore; 4 Departments of Epidemiology, Biostatistics & Occupational Health and of Pediatrics, McGill University Faculty of Medicine, Montreal, QC, Canada; 5 Department of Epidemiology, School of Public Health, University of Pittsburgh, Pittsburgh, PA, United States of America; Rotunda Hospital, IRELAND

## Abstract

**Background:**

Women are less likely to have classic cardiovascular risk factors than men, and events during their reproductive and menopausal years may increase hypertension risk. The aim of this study is to examine woman-specific factors, including menstrual, reproductive and pregnancy complications, in relation to the prevalence of hypertension in mid-life Asian women.

**Methods:**

This is a cross-sectional study of 1146 healthy women aged 45–69 years, from a multi-ethnic Asian cohort. The women completed an extensive questionnaire that included their sociodemographic details, medical history, lifestyle and physical activity, and reproductive and menopausal history. They also underwent objectively measured physical performance tests and a dual X-ray absorptiometry scan. Hypertension was defined as a systolic BP ≥140 and/or diastolic BP ≥90mm Hg, past diagnosis by a physician, or use of antihypertensive medications. Multivariable logistic regression was used to assess the independent risk factors for hypertension.

**Results:**

The average age of the 1146 women analysed was 56.3 (SD 6.2) years, and 55.2 percent of them were hypertensive. The prevalence of gestational diabetes and gestational hypertension was 12.6% and 9.4%, respectively. Besides age, abnormal menstrual cycle length at 25 years of age (OR:2.35, CI:1.34–4.13), preeclampsia (OR:2.46, CI:1.06–5.74), increased visceral adiposity (OR:4.21, CI:2.28–7.79) and reduced physical performance (OR:2.83, CI:1.46–5.47) were independently associated with hypertension in Asian women.

**Conclusions:**

Our findings highlight the necessity of including features of menstrual and reproductive history as possible indicators of hypertension risk in cardiovascular disease risk assessment and prevention among Asian women. Reducing visceral adiposity and exercise to improve physical performance may help women avoid developing hypertension.

## Introduction

Hypertension is a major cause of cardiovascular disease (CVD), leading to 1 in 3 deaths in women globally [1]. The prevalence of hypertension continues to rise in Asia [2] and in other rapidly aging populations [3]. The sequelae of hypertension, such as stroke, chronic kidney disease, and ischemic heart disease, increasingly affect mid-life women [4, 5].

Men and women differ in the development of hypertension [6]. Besides established risk factors such as physical inactivity, high sodium diet, and obesity, sex-specific factors across a woman’s life course may affect her risk of developing hypertension [4]. Reproductive factors such as gestational hypertension, preeclampsia, and miscarriages are underpinned by endothelial dysfunction, which may predispose women to hypertension [7]. The duration of breastfeeding, the number of live births, premature ovarian failure, or early onset menopause have also been linked to the risk of hypertension [8, 9]. While premenopausal women have a lower prevalence of hypertension than men, the rates of hypertension increase with age in both sexes. As women transition through menopause, the rate of hypertension rises steeply and can exceed that of men around the sixth decade of life [4]. This menopausal transition, whereby ovarian reserves and oestrogen levels decline, precedes the subsequent acceleration of hypertension and other CVD risk factors [5]. Most studies on menopause and other female-specific factors have focused on Caucasian populations. Few studies have been published on Asian mid-life women, particularly those of Chinese ethnicity. Our cohort of multi-ethnic women from Singapore is ideally placed to explore these factors. In our prior research that investigated the association of visceral adiposity with inflammatory markers as mediators of systolic blood pressure in middle-aged women, we noticed a trend, albeit insignificant, towards lower systolic blood pressure with the presence of vasomotor symptoms. However, the study was not designed to and did not thoroughly investigate reproductive risk factors [10].

This study examines woman-specific factors, such as menstrual history, reproductive history, pregnancy complications, menopausal symptoms, anthropometric measures, and upper- and lower-body muscle strength in relation to the prevalence of hypertension in mid-life Asian women. Identifying such woman-specific markers is important because many women do not have traditional risk factors for hypertension, and the early detection of hypertension can reduce the burden of CVD.

## Methods

### Population

Healthy women aged 45 to 69 years who were attending the outpatient gynaecological clinics at the National University Hospital, Singapore, for routine perimenopausal care were invited to enrol in the integrated women’s health program (IWHP), a multi-ethnic Asian cohort of midlife women. Posters and leaflets were placed in the outpatient clinics and at public events organized by the hospital. Clinic personnel also notified eligible patients about the study and contacted the recruiting coordinators [11]. Women who were currently pregnant, with a history of cancer or other potentially life-threatening conditions, such as end stage renal or liver disease, or an anticipated life expectancy of less than a year, were excluded from this study. From 2014 to 2016, a total of 2175 women were screened. However, 746 declined to participate, 134 failed to attend the appointment, and 110 could not be contacted, resulting in a final sample size of 1201 women. The detailed study methodology of the IWHP cohort has been previously described [11]. The recording of the baseline cohort data began in 2014 and collection completed by 2017. The data was accessed for this study from June 2022 to April 2023. As of the submission of this manuscript, participants are returning for their first follow-up visit. The study received ethics approval from the Domain Specific Review Board of the National Healthcare Group, Singapore (reference number 2014/00356). All participants provided written informed consent. This study adhered to the items required in the STROBE statement [12].

### Data collection and study measurements

Participants completed a comprehensive self-administered questionnaire encompassing their sociodemographic, past medical history, cardiovascular risk factors, drug history, lifestyle and physical activity, quality of life indicators, and detailed reproductive health and menopausal history. The past medical history was based on physician diagnoses. Current medication use was assessed by asking participants to bring all medications to the enrolment interview. Questions on reproductive and menopausal history were adapted from the Study of Women Across the Nation (SWAN) [13]. These included a detailed menstrual history, pregnancy history, menopausal history, and symptoms.

In the reproductive history section, women were asked about previous pregnancies, breastfeeding, and complications, such as preeclampsia, gestational diabetes, and miscarriages. Prior menstrual history at 25 years of age was considered regular if cycle lengths were within 21–35 days and irregular if it was <21 or >35 days. Menopause-related history included hot flashes, palpitations, sleep, mood, genitourinary symptoms, and musculoskeletal symptoms based on the Menopause Rating Scale (MRS) [14]. Pre-menopause was defined as regular menstruation with no change in menstrual cycles during the past 12 months, with the most recent period occurring within the last three months. Perimenopause was defined as a change from the baseline menstrual frequency or amenorrhea for less than 12 months. Menopause was defined as amenorrhea for more than 12 consecutive months or surgical menopause.

After completing the questionnaire, the participants underwent a physical examination conducted by trained study personnel. The height and weight of the women were measured using the SECA 769 electronic station. Body mass index was calculated as weight (kg) divided by height squared (kg/m^2^). Participants underwent a short physical performance battery test (SPPB) [15]. Lower extremity strength was assessed by the time taken to complete five chair stands. Upper-extremity handgrip strength was measured using the Jamar hand dynamometer. To assess the body composition parameters of appendicular lean body mass (ALM) and visceral adipose tissue (VAT) accurately, the dual X-ray absorptiometry (DXA) scanner was used (Hologic Discovery Wi Apoex software 4.5). VAT was split into tertiles; lower (<88.6 cm^2^), middle (88.6-131cm^2^), higher (>131cm^2^).

The outcome variable of hypertension was defined as (i) systolic BP ≥140 and/or diastolic BP ≥90mm Hg, (ii) past diagnosis of hypertension by a healthcare professional, or (iii) use of antihypertensive medications. Blood pressure was measured with an appropriately sized arm cuff using the OMRON Intellisense meter (HEM7211). Three separate readings were obtained in a seated position after 5 minutes of rest and separation of one-minute intervals between each reading. The average blood pressure was calculated as the mean of three separate readings obtained at the outpatient clinics. All variables in this study were collected at the baseline visit of this cohort. All data used in the analysis were de-identified.

### Statistical analysis

In this cross-sectional analysis, baseline characteristics of the women were analysed as numbers and percentages for categorical variables and means and standard deviations for continuous variables. We assessed crude (unadjusted) associations of potential risk factors for hypertension, including sociodemographic and lifestyle characteristics, reproductive risk factors, menopausal symptoms, and anthropometric and physical performance measures, which were compared using the chi-square test for categorical variables and Student’s t-test for continuous variables. Subsequently, multiple logistic regression was then used to assess independent predictors of hypertension. Hypertension was considered as a dichotomous dependent variable, whereas traditional risk factors, menopausal symptoms, and reproductive factors were included as independent variables. The final adjusted model was adjusted for sociodemographic factors, lifestyle characteristics encompassing age, ethnicity, education, employment, smoking and alcohol history, diabetes, and use of hormone replacement therapy. Reproductive factors included age at menarche, previous menstrual cycle length, breast lumps, marital status, pregnancy-related factors (parity, miscarriage, stillbirth, breastfeeding history, preeclampsia, and gestational diabetes), menopausal status, and history of hysterectomy and/or bilateral oophorectomy. Anthropometric and physical performance measures included height, VAT, short performance battery test, and handgrip strength. As a hierarchical regression revealed very similar results, all independent variables were included in the reported model results, except for BMI and diabetes, which were excluded because of their high collinearity with VAT and gestational diabetes. All statistical analyses were conducted using SPSS version 25 (Chicago, IL, USA), with statistical significance set at 2-sided p<0.05.

## Results

### Participant characteristics

Of the 1,201 women enrolled, we analysed the data of 1,146 women after excluding 13 women who had incomplete data on blood pressure and 42 women whose ethnicity was not recorded as Chinese, Malay, or Indian. The participant characteristics are described in **Table 1**. The mean age was 56.3 years, with an SD of 6.2 years. More than 80% of the cohort women were Chinese, 40.2% had received tertiary education or equivalent, and more than half were employed. Four-fifths were married, 1.9% were smokers, and 3.1% drank more than one unit of alcohol per week. Of the 1,146 women analysed, 55.2% (CI 0.52, 0.58) were hypertensive.

**Table 1:** Characteristics of study participants (n = 1146).

	n (%)	Hypertension (n = 633)	No Hypertension (n = 513)	*p*-value^a^
**Socio-demographics & Lifestyle**				
Ethnicity				0.348
Chinese	963 (84.0)	523 (54.3)	440 (45.7)	
Malay	64 (5.6)	38 (59.4)	26 (40.6)	
Indian	119 (10.4)	72 (60.5)	47 (39.5)	
Age (years), mean (SD)				<0.001
45–54, 50.6 (2.7)	496 (43.3)	220 (44.4)	276 (55.6)	
55–64, 59.1 (2.8)	505 (44.1)	313 (62.0)	192 (38.0)	
≥ 65, 66.7 (1.3)	144 (12.6)	100 (69.4)	44 (30.6)	
Education				<0.001
University	457 (40.2)	221 (48.4)	236 (51.6)	
Secondary	512 (45.1)	306 (59.8)	206 (40.2)	
No formal or primary	167 (14.7)	102 (61.1)	65 (38.9)	
Employment				0.004
Yes	770 (67.3)	403 (52.3)	367 (47.7)	
No	374 (32.7)	228 (61.0)	146 (39.0)	
Monthly Household Income (SGD)				0.058
Lower (<3000)	286 (28.6)	168 (58.7)	118 (41.3)	
Higher (≥3000)	713 (71.4)	370 (51.9)	343 (48.1)	
Smoking				0.392
Yes	22 (1.9)	10 (45.5)	12 (54.5)	
No	1122 (98.1)	621 (55.3)	501 (44.7)	
Alcohol intake				0.491
Yes	35 (3.1)	17 (48.6)	18 (51.4)	
No	1105 (96.9)	612 (55.4)	493 (44.6)	
Diabetes				<0.001
Yes	288 (25.1)	234 (81.3)	54 (18.8)	
No	858 (74.9)	399 (46.5)	459 (53.5)	
**Women-specific correlates**				
Marital status				0.820
Married	928 (81.1)	511 (55.1)	417 (44.9)	
Not married	216 (18.9)	121 (56.0)	95 (44.0)	
Parity				0.744
Nulliparous	197 (17.2)	109 (55.3)	88 (44.7)	
1 or 2 children	642 (56/0)	360 (56.1)	282 (43.9)	
3 or more children	307 (26.8)	164 (53.4)	143 (46.6)	
Age of menarche (years), mean (SD)	13.1 (1.5)	13.1 (1.5)	13.2 (1.5)	0.227
Menstrual cycle regularity				0.011
Regular	957 (84.2)	514 (53.7)	443 (46.3)	
Irregular	179 (15.8)	115 (64.2)	64 (35.8)	
Menstrual cycle length				0.439
Normal	747 (81.9)	396 (53.0)	351 (47.0)	
Abnormal	165 (18.1)	93 (56.4)	72 (43.6)	
Miscarriage				0.463
Yes	364 (37.5)	197 (54.1)	167 (45.9)	
No	607 (62.5)	344 (56.7)	263 (43.3)	
Stillbirth				0.544
Yes	46 (4.7)	28 (60.9)	18 (39.1)	
No	924 (95.3)	512 (55.4)	412 (44.6)	
Breastfeeding				0.016
Yes	652 (67.3)	343 (52.6)	309 (47.4)	
No	317 (32.7)	193 (60.9)	124 (39.1)	
Gestational diabetes				0.011
Yes	119 (12.6)	79 (66.4)	40 (33.6)	
No	829 (87.4)	448 (54.0)	381 (46.0)	
Gestational hypertension				<0.001
Yes	89 (9.4)	70 (78.7)	19 (21.3)	
No	858 (90.6)	454 (52.9)	404 (47.1)	
Breast lump/ cysts				0.725
Yes	150 (13.5)	81 (54.0)	69 (46.0)	
No	962 (86.5)	535 (55.6)	427 (44.4)	
Hysterectomy				0.766
Yes	124 (14.6)	76 (61.3)	48 (38.7)	
No	725 (85.4)	431 (59.4)	294 (40.6)	
Oophorectomy				0.595
Yes	97 (8.5)	51 (52.6)	46 (47.4)	
No	1040 (92.5)	577 (55.5)	463 (44.5)	
Menopausal status				<0.001
Pre-menopausal	146 (12.7)	59 (40.4)	87 (59.6)	
Peri-menopausal	176 (15.4)	80 (45.5)	96 (54.5)	
Post-menopausal	824 (71.9)	494 (60.0)	330 (40.0)	
Age at menopause (years), Mean (SD)	49.7 (4.4)	49.9 (4.4)	49.4 (4.4)	0.093
Menopausal hormone therapy^b^				0.161
Yes	19 (1.7)	14 (73.7)	5 (26.3)	
No	1127 (98.3)	619 (54.9)	508 (45.1)	
**Anthropometry and physical performance**				
Body mass index (kg/m^2^)				<0.001
Normal or underweight, <23.0	539 (47.0)	253 (46.9)	286 (53.1)	
Overweight, 23.0–27.49	395 (34.5)	228 (57.7)	167 (42.3)	
Obese, ≥27.5	212 (18.5)	152 (71.7)	60 (28.3)	
Visceral adipose tissue				<0.001
Lowest tertile	382 (33.6)	146 (38.2)	236 (61.8)	
Middle tertile	379 (33.3)	213 (56.2)	166 (43.8)	
Highest tertile	376 (33.1)	269 (71.5)	107 (28.5)	
Height in cm, mean (SD)	157.1 (8.9)	156.8 (10.9)	157.3 (5.4)	0.390
Physical performance^d^				<0.001
High (SPPB >10)	959 (83.7)	504 (52.6)	455 (47.4)	
Moderate or low (SPPB ≤10)	187 (16.3)	129 (69.0)	58 (31.0)	
Average handgrip strength (kg), mean (SD)	17.7 (6.5)	17.5 (6.4)	18.0 (6.4)	0.264
ALM (kg/m2), mean (SD) §	5.7 (3.0)	5.8 (3.2)	5.5 (2.9)	0.080

ALM: Appendicular lean mass, SD: standard deviation, SGD: Singaporean dollars, SPPB: Short physical performance battery

^a^ Associations with hypertensive status were assessed with chi-square tests or t-tests where appropriate

^b^ Systemic MHT excluding local/topical oestrogen

^c^ Physical performance by Short Physical Performance Battery

^d^ Appendicular lean muscle mass

At the time of enrolment into the study, 12.7% were premenopausal, 15.4% perimenopausal, 71.9% were postmenopausal, and 17.2% were nulliparous. Of the 971 women with previous pregnancies, 37.5% experienced a prior miscarriage, 46 of which occurred after the 20^th^ week of gestation. Among the mothers, 67.3% breastfed their children. Among those with a previous pregnancy, the prevalence rate of gestational diabetes was 12.6%, and gestational hypertension was 9.4%. At the age of 25 years, 15.8% of the women had a history of irregular menstrual periods. Only 1.7% of the women received systemic menopausal hormone therapy. Antihypertensive medications were consumed by 40.4% of women.

Crude associations between hypertension status and sociodemographic and lifestyle characteristics, reproductive risk factors, menopausal symptoms, and anthropometric and physical risk factors are shown in **Table 1**. Older women, lower educational levels, unemployment, and diabetes were associated with hypertension, whereas no association was observed for ethnicity, monthly household income, smoking, and alcohol consumption. As expected, older age was highly correlated with hypertension; before adjustment, women aged 55–64 years had twofold higher odds of hypertension, whereas women aged 65 years and older had fourfold higher odds of hypertension than those aged 45–54. Among the reproductive risk factors, irregular menstrual cycle length, lack of history of breastfeeding in a previous pregnancy, gestational diabetes, gestational hypertension, and postmenopausal status were significantly associated with hypertension. Overweight and obese women had a significantly higher prevalence of hypertension than normal or underweight women. The middle and highest VAT tertiles, moderate or low physical performance, and longer time to complete the repeated chair-stand test were associated with hypertension, whereas the average handgrip strength and appendicular lean muscle mass were not.

**Table 2:** Adjusted odds ratios (and 95% CI) for hypertension in midlife Singaporean women based on multivariable logistic regression.

Variables	adjusted OR (95% CIs)
**Socio-demographics & Lifestyle**	
Ethnicity	
Chinese	Ref
Malay	1.11[0.38,3.25]
Indian	0.93[0.47,1.85]
Age (years)	
45–54	Ref
55–64	1.63[0.98,2.70]
≥ 65	3.39[1.55,7.39]
Education	
University	0.60[0.29,1.24]
Secondary	0.91[0.46,1.80]
No formal or primary	Ref
Employment	
Yes	1.14 [0.71, 1.82]
No	Ref
Monthly Household Income (SGD)	
Higher (>3000)	0.91 [0.55, 1.52]
Lower (<3000)	Ref
Smoking	
Yes	1.23 [0.26, 5.81]
No	Ref
Alcohol intake	
Yes	1.67 [0.44, 6.34]
No	Ref
**Women-specific correlates**	
Marital status	
Married	1.28 [0.65, 2.49]
Not married	Ref
Parity	
Nulliparous	Ref
1 or 2 children	0.47[0.12,1.85]
3 or more children	0.28[0.07,1.16]
Age of menarche (years), mean (SD)	1.02 [0.87, 1.20]
Menstrual cycle length	
Normal	Ref
Abnormal	2.35[1.34,4.13]
Miscarriage	
Yes	0.84[0.54,1.32]
No	Ref
Stillbirth	
Yes	1.37[0.40,4.64]
No	Ref
Breastfeeding	
Yes	1.30[0.79,2.12]
No	Ref
GDM	
Yes	1.96[0.97,3.95]
No	Ref
Pre-eclampsia	
Yes	2.46[1.06,5.74]
No	Ref
Breast lump/ cysts	
Yes	0.69[0.39,1.22]
No	Ref
Hysterectomy	
Yes	1.83 [0.80, 4.16]
No	Ref
Oophorectomy	
Yes	0.67 [0.26, 1.71]
No	Ref
Menopausal status	
Pre-menopausal	Ref
Peri-menopausal	2.90 [0.34, 24.45]
Post-menopausal	2.74 [0.44, 15.56]
**Anthropometry and physical performance**	
Visceral adipose tissue	
Lowest tertile	Ref
Middle tertile	1.99[1.18,3.33]
Highest tertile	4.21[2.28,7.79]
Height (cm)	0.95[0.91,0.99]
Physical performance^a^	
High (SPPB >10)	Ref
Moderate or low (SPPB ≤10)	2.83[1.46,5.47]
Average handgrip strength (kg)	1.03[0.99,1.06]
ALM mass (kg/m2)	1.12[0.84,1.51]

ALM: Appendicular lean mass, SD: standard deviation, SGD: Singaporean dollars, SPPB: Short physical performance battery

^a^ Physical performance by Short Physical Performance Battery

Menopausal hormone therapy was excluded in the model due to insufficient observations (n = 19)

Reference groups (Ref) were chosen to provide clinically relevant differences with adequate statistical power as well as to be consistent with other categories used in the IWHP cohort to explore other outcomes.

Among reproductive factors, women who reported abnormal menstrual cycle length had a 2.35-fold (1.34–4.13) increased risk of hypertension compared to women with regular menstrual cycles. Women with a history of preeclampsia were also at increased risk (aOR 2.46 [1.06–5.74]). While gestational diabetes appeared to have a trend toward an increased risk of hypertension, the association was statistically not significant (aOR 1.96 [0.97–3.95]). Women with poor physical performance as measured with SPPB, were at a 2.83-fold [1.46–5.47] higher risk. The strongest association was observed with visceral adiposity, wherein women in the highest tertile had a four-fold (aOR 4.21 [2.28–7.79]) higher risk than those in the lowest tertile.

## Discussion

To our knowledge, this is the most extensive multi-ethnic Asian study that details the association between reproductive risk factors and hypertension with well-validated measurement methods and tools such as the DXA scan for ALM and VAT, rather than surrogate measures. Our findings identified irregular menstrual periods, VAT, reduced physical performance, and preeclampsia as significant reproductive risk factors for hypertension.

While it is well established that aging is a significant risk factor for hypertension, the marked increase in the risk of hypertension among IWHP women aged 65 years and older was higher than expected. In a comparative study of hypertension prevalence among adults in China and Sweden, Santosa et al. also found that the prevalence of hypertension was higher among mid-life Chinese women compared to Swedish women [16]. The onset of hypertension may begin earlier in Asian or eastern ethnicities, and preventive measures and screening should be started earlier to prevent complications of prolonged exposure to untreated hypertension.

Another key finding was that women with a history of preeclampsia had an almost two and a half-fold increase in the prevalence of hypertension. Preeclampsia is a recognized risk factor for hypertension in the latest guideline update on CVD prevention in women by the American College of Cardiology [17]. Our findings are also in agreement with multiple studies and a previous meta-analysis of 1885 women with preeclampsia, which reported a 3.7 times higher risk of developing hypertension in life [18]. The higher reported risk could be due to the longer follow-up period of approximately 14 years. Longitudinal follow-up of our cohort may show a similar pattern. Although it is widely accepted that preeclampsia and hypertension are linked due to the shared pathophysiology of endothelial dysregulation and increased systemic vascular resistance, resulting in elevated blood pressure, the exact mechanism underlying the link between preeclampsia and future hypertension remains unknown [19]. Questions persist as to whether preeclampsia causes cardiovascular and metabolic alterations during pregnancy that then cause the development of hypertension later in life or whether the association is due to common risk factors.

In our cohort, we observed that a past history of abnormal menstrual cycle lengths at 25 years of age, both short (<21 days) and long (>35 days), was significantly independently associated with future hypertension. While abnormal menstrual cycles may predispose women to future metabolic disorders and CVD [20], few studies have explicitly examined the relationship between menstrual cycle length and hypertension. A recent study evaluating premenstrual symptoms in Australian women found a non-significantly higher incidence of hypertension in women with painful and irregular periods [21]. In another prospective study analysing irregular periods and metabolic disorders in Iranian women, Dovom et al. observed that irregular periods were associated with diabetes but not with the incidence rates of dyslipidaemia or hypertension. However, irregular periods were defined only as having long cycles of more than 35 days. Short menstrual cycles were not differentiated from the average cycle length. It is thought that some of the menstrual irregularities could be due to polycystic ovarian syndrome (PCOS). Women with PCOS are characterized by features of hyperandrogenism, oligomenorrhea, and oligomenorrhea or amenorrhea in women of reproductive age. PCOS is also associated with a higher prevalence of elevated BMI, insulin resistance, and elevated blood pressure [22]. Notwithstanding, PCOS is more commonly associated with longer cycle lengths and does not explain the association we observed with hypertension even with shorter menstrual cycle lengths. A simple question about menstrual cycle length in young women could be a valuable tool for screening for future hypertension risk. Further studies are required to investigate the association between menstrual cycle irregularities and hypertension.

We did not confirm our hypothesized association between vasomotor symptoms of menopause and hypertension, both of which are underpinned by endothelial dysfunction. Previous studies have been heterogeneous, with the inclusion of women on hormone replacement therapy, relying on self-reporting of CVD risk factors and differing definitions of hypertension [23–26]. The absence of an association is consistent with the results of studies conducted in Asia, including Korean and Chinese studies evaluating vasomotor symptoms and metabolic syndrome. Both studies showed that vasomotor symptoms were associated with metabolic syndrome but not with hypertension alone [27, 28]. Our study further adds to the evidence that vasomotor symptoms in Asian women are not associated with hypertension.

Beyond reproductive factors, we found that higher VAT and lower SPPB scores were associated with the prevalence of hypertension. The relationship between high VAT and hypertension is well-established and consistent with our findings [29]. Details regarding the association between visceral adiposity and inflammatory markers as mediators of systolic blood pressure have been published [10].

The SPPB protocol is a series of tests designed to evaluate physical performance and has been utilized extensively to assess lower extremity function, balance, and mobility in older populations. Recent publications by Bellettiere et al. analysing older women (mean age 79 ± 7 years) revealed that lower SPPB scores were associated with an increased risk of incident acute heart failure and cardiovascular events [30, 31]. Specifically, hypertension attenuated the association between SPPB scores and heart failure but did not affect their overall significance. Although the average age of the women in our population was younger (mean age 56.3± 6.2 years), low to moderate SPPB scores correlated with hypertension. While it is well established that exercise is essential for the prevention and management of hypertension, the ideal form of exercise regimen for preventing or managing blood pressure has not yet been determined [32, 33]. Further research is required to determine the type of exercise that improves SPPB components and total physical performance in women. In addition to postmenopausal women, the SPPB test may also be beneficial for determining the risk of cardiovascular disease in younger perimenopausal women.

Women appear to have two distinct life-course phases that affect their cardiovascular system: pregnancy and menopause. Few previous studies have focused on menstrual history and CVD risk or hypertension. Future studies and longitudinal data we are currently collecting from the IWHP cohort will help us to better understand the reproductive risks of hypertension.

### Study limitations

Our study has several limitations. First, it is cross-sectional and therefore cannot determine temporality and causality. However, most of the reproductive risk factors we studied usually predate the onset of hypertension. Also, despite meticulous attempts to phenotype our cohort, we cannot exclude recall bias and over- or underreporting variables such as previous menstrual cycle history trend and menopausal symptoms. Nevertheless, the menstrual cycle history was based around age 25, when the hypothalamus-pituitary-ovarian axis would have reached maturity and cycles would often be regular in most women [34]. The women were informed to provide their menstrual cycle history based on the year after if they were pregnant or breast feeding. Other cohorts such as the SWAN have used similar questionnaires to analyse menstrual characteristics [35], and the MRS tool that we used is widely accepted as the standard for assessing menopausal symptoms in studies. While we attempted to be comprehensive in the data collection of our cohort, additional unaccounted confounders such as hyperlipidaemia history and dietary habits were not measured. Another limitation of the cohort is that women were recruited from one gynecological clinic. Singapore is a city-state with a large population concentrated in a small geographic area. The study hospital attracts healthy women for its comprehensive screening and menopausal services, as well as for tertiary care. Nonetheless, recruitment of our study cohort was not based on random sampling, and our results may not be generalizable to the Singapore population. Nevertheless, women in the study are comparable to that of Singapore’s general population in many aspects [36], 81% were of Chinese ethnic origin (vs 80% in the general population); 40% had post-secondary education (vs 38%), and 67% were employed (vs 60%). However, the findings may not be generalisable to populations in the west. Nevertheless, our findings are likely to be relevant to other Chinese predominant populations.

## Conclusion

Our findings in this multi-ethnic cross-sectional study of Asian women indicate that abnormal length of menstrual cycles, pre-eclampsia, elevated visceral adiposity, and poor physical performance are strongly associated with hypertension. The findings underscore that CVD risk assessment and prevention in women should include aspects of their menstrual and reproductive history as potential indicators of risk for hypertension. Prospective longitudinal follow-up of our study women will continue to provide further insights into female sex-specific risk factors of hypertension. Two of the identified risk factors, namely visceral adiposity, and poor physical performance, are modifiable. Public health efforts to reduce obesity and increase exercise are urgently required to reduce the high prevalence of hypertension and its adverse sequelae in mid-life women.

## Supporting information

S1 Checklist (DOCX)
