# Supplementary material for: Associations between hypertension with reproductive and menopausal factors: An integrated women’s health programme (IWHP) study
Source: PLoS One. 2024 Mar 25;19(3):e0299840. doi: 10.1371/journal.pone.0299840 (PMC10962834; doi:10.1371/journal.pone.0299840)
Supplement: S1 Checklist — (DOCX) [file pone.0299840.s001.docx]

STROBE Statement—checklist of items that should be included in reports of observational studies

|  | Item No. | Recommendation | Page  No. | Relevant text from manuscript |
| --- | --- | --- | --- | --- |
| **Title and abstract** | 1 | (*a*) Indicate the study’s design with a commonly used term in the title or the abstract | 1, 2 | “Associations between hypertension with reproductive and menopausal factors:  An Integrated Women’s Health Programme (IWHP) study” |
|  |  | (*b*) Provide in the abstract an informative and balanced summary of what was done and what was found | 2 | “This is a cross-sectional study..” |
| Introduction | | | |  |
| Background/rationale | 2 | Explain the scientific background and rationale for the investigation being reported | 4, 5 | “Identifying such woman-specific markers is important because many women do not have conventional risk factors, and the early detection of hypertension can reduce the burden of CVD.” |
| Objectives | 3 | State specific objectives, including any prespecified hypotheses | 5 | “This study examines woman-specific factors, such as menstrual history, reproductive history, pregnancy complications, menopausal symptoms, anthropometric measures, and upper- and lower-body muscle strength in relation to the prevalence of hypertension in mid-life Asian women” |
| Methods | | | |  |
| Study design | 4 | Present key elements of study design early in the paper | 5,6,7 | Methods – population, data collection and study measurements, and statistical analysis |
| Setting | 5 | Describe the setting, locations, and relevant dates, including periods of recruitment, exposure, follow-up, and data collection | 5,6,7 | Methods – data collection and study measurements |
| Participants | 6 | (*a*) *Cohort study*—Give the eligibility criteria, and the sources and methods of selection of participants. Describe methods of follow-up  *Case-control study*—Give the eligibility criteria, and the sources and methods of case ascertainment and control selection. Give the rationale for the choice of cases and controls  *Cross-sectional study*—Give the eligibility criteria, and the sources and methods of selection of participants | 5 | “Women who were currently pregnant, with a history of cancer or other potentially life-threatening conditions were excluded from this study.”  “Healthy women aged 45 to 69 years who were attending the outpatient gynaecological clinics at the National University Hospital, Singapore, for routine perimenopausal care were invited to enrol in the integrated women’s health program (IWHP)" |
|  |  | (*b*) *Cohort study*—For matched studies, give matching criteria and number of exposed and unexposed  *Case-control study*—For matched studies, give matching criteria and the number of controls per case |  |  |
| Variables | 7 | Clearly define all outcomes, exposures, predictors, potential confounders, and effect modifiers. Give diagnostic criteria, if applicable | 5,6,7, 12,13 | Methods – data collection and study measurements.  And adjusted odds ratio table 2 |
| Data sources/ measurement | 8* | For each variable of interest, give sources of data and details of methods of assessment (measurement). Describe comparability of assessment methods if there is more than one group | 5,6,7 | Methods – data collection and study measurements |
| Bias | 9 | Describe any efforts to address potential sources of bias | 17,18 | Study limitations |
| Study size | 10 | Explain how the study size was arrived at | 5 | “From 2014 to 2016, a total of 2175 women were screened. However, 746 declined to participate, 134 failed to attend the appointment, and 110 could not be contacted, resulting in a final sample size of 1201 women.” |

Continued on next page

| Quantitative variables | 11 | Explain how quantitative variables were handled in the analyses. If applicable, describe which groupings were chosen and why | 5,6,7,8 | See section on Methods- data collection and study measurements, and statistical analysis |
| --- | --- | --- | --- | --- |
| Statistical methods | 12 | (*a*) Describe all statistical methods, including those used to control for confounding | 7,8 | See section on Methods – statistical analysis |
|  |  | (*b*) Describe any methods used to examine subgroups and interactions | 7,8 | See section on Methods – statistical analysis |
|  |  | (*c*) Explain how missing data were addressed | 8 | “Of the 1,201 women enrolled, we analysed the data of 1,146 women after excluding 13 women who had incomplete data on blood pressure and 42 women whose ethnicity was not recorded as Chinese, Malay, or Indian.” |
|  |  | (*d*) *Cohort study*—If applicable, explain how loss to follow-up was addressed  *Case-control study*—If applicable, explain how matching of cases and controls was addressed  *Cross-sectional study*—If applicable, describe analytical methods taking account of sampling strategy |  |  |
|  |  | (*e*) Describe any sensitivity analyses | 7 | “The final adjusted model was adjusted for sociodemographic factors, lifestyle characteristics encompassing age, ethnicity, education, employment, smoking and alcohol history, diabetes, and use of hormone replacement therapy” |
| Results | | | | |
| Participants | 13* | (a) Report numbers of individuals at each stage of study—eg numbers potentially eligible, examined for eligibility, confirmed eligible, included in the study, completing follow-up, and analysed | 5, 8 | “From 2014 to 2016, a total of 2175 women were screened. However, 746 declined to participate, 134 failed to attend the appointment, and 110 could not be contacted, resulting in a final sample size of 1201 women.” |
|  |  | (b) Give reasons for non-participation at each stage | 5 | “From 2014 to 2016, a total of 2175 women were screened. However, 746 declined to participate, 134 failed to attend the appointment, and 110 could not be contacted, resulting in a final sample size of 1201 women.” |
|  |  | (c) Consider use of a flow diagram | 5 | “The detailed study methodology of the IWHP cohort has been previously described “ |
| Descriptive data | 14* | (a) Give characteristics of study participants (eg demographic, clinical, social) and information on exposures and potential confounders | 8,9,10 | See section on Results – participant characteristics |
|  |  | (b) Indicate number of participants with missing data for each variable of interest | 8 | “Of the 1,201 women enrolled, we analysed the data of 1,146 women after excluding 13 women who had incomplete data on blood pressure and 42 women whose ethnicity was not recorded as Chinese, Malay, or Indian.” |
|  |  | (c) *Cohort study*—Summarise follow-up time (eg, average and total amount) |  |  |
| Outcome data | 15* | *Cohort study*—Report numbers of outcome events or summary measures over time |  |  |
|  |  | *Case-control study—*Report numbers in each exposure category, or summary measures of exposure | 8,9,10 | Table 1 |
|  |  | *Cross-sectional study—*Report numbers of outcome events or summary measures |  |  |
| Main results | 16 | (*a*) Give unadjusted estimates and, if applicable, confounder-adjusted estimates and their precision (eg, 95% confidence interval). Make clear which confounders were adjusted for and why they were included | 8,9,10,13,14 | Table 1 and 2 |
|  |  | (*b*) Report category boundaries when continuous variables were categorized | 8,9,10,13,14 |  |
|  |  | (*c*) If relevant, consider translating estimates of relative risk into absolute risk for a meaningful time period |  |  |

Continued on next page

| Other analyses | 17 | Report other analyses done—eg analyses of subgroups and interactions, and sensitivity analyses |  |  |
| --- | --- | --- | --- | --- |
| Discussion | | | | |
| Key results | 18 | Summarise key results with reference to study objectives | 15 | “Our findings identified irregular menstrual periods, VAT, reduced physical performance, and preeclampsia as significant reproductive risk factors for hypertension.” |
| Limitations | 19 | Discuss limitations of the study, taking into account sources of potential bias or imprecision. Discuss both direction and magnitude of any potential bias | 18,19 | See section on study limitations |
| Interpretation | 20 | Give a cautious overall interpretation of results considering objectives, limitations, multiplicity of analyses, results from similar studies, and other relevant evidence | 15, 16, 17, 18 | Discussion section. |
| Generalisability | 21 | Discuss the generalisability (external validity) of the study results | 19 | “Our findings may not be generalisable to populations in the west. Nevertheless, our findings are likely to be relevant to other Chinese predominant populations.” |
| Other information | |  | | |
| Funding | 22 | Give the source of funding and the role of the funders for the present study and, if applicable, for the original study on which the present article is based | 20 | “Development of this study cohort was partially funded by the Singapore National Medical Research Council Grant (Reference number: NMRC/CSA-SI/0010/2017)” |

*Give information separately for cases and controls in case-control studies and, if applicable, for exposed and unexposed groups in cohort and cross-sectional studies.

**Note:** An Explanation and Elaboration article discusses each checklist item and gives methodological background and published examples of transparent reporting. The STROBE checklist is best used in conjunction with this article (freely available on the Web sites of PLoS Medicine at http://www.plosmedicine.org/, Annals of Internal Medicine at http://www.annals.org/, and Epidemiology at http://www.epidem.com/). Information on the STROBE Initiative is available at www.strobe-statement.org.
